# Prevalence of Hormonal Contraceptive Use and Self‐Reported Symptomatic Experiences Attributed to the Menstrual Cycle or Hormonal Contraceptive Use in Norwegian Women: The Effect of Training Categories and Age Groups ‐ The FENDURA Project

**DOI:** 10.1111/sms.70096

**Published:** 2025-07-12

**Authors:** John Owen Osborne, Jonas H. Storvand, Tina P. Engseth, Guro S. Solli, Bente Morseth, Madison Y. Taylor, Boye Welde, Kirsty J. Elliott‐Sale, Erik P. Andersson, Øyvind Sandbakk, Dionne A. Noordhof

**Affiliations:** ^1^ School of Sport Sciences UiT the Arctic University of Norway Tromsø Norway; ^2^ Sunshine Coast Hospital and Health Service Birtinya Queensland Australia; ^3^ School of Health University of the Sunshine Coast Sippy Downs Queensland Australia; ^4^ School of Exercise and Nutrition Sciences Queensland University of Technology Brisbane Queensland Australia; ^5^ Department of Sports Science and Physical Education Nord University Bodø Norway; ^6^ Division of Public Health and Prevention, Department of Child and Adolescent Health Promotion Services Norwegian Institute of Public Health Levanger Norway; ^7^ Department of Sport and Exercise Sciences Manchester Metropolitan University Institute of Sport Manchester UK; ^8^ Department of Health Sciences, Swedish Winter Sports Research Centre Mid Sweden University Östersund Sweden; ^9^ Centre for Elite Sports Research, Department of Neuromedicine and Movement Science Norwegian University of Science and Technology Trondheim Norway

**Keywords:** combined oral contraceptive, long‐acting reversible contraception, menstrual disorders, progestin‐only contraceptives, symptoms

## Abstract

The aims of the current study were to (1) report the prevalence of hormonal contraceptive (HC) use among Norwegian women across different training categories and age groups; (2) compare the frequency and severity of cycle‐related symptoms across differing training categories and age groups; and (3) describe the bleeding pattern and prevalence of menstrual disorder/disturbances among non‐HC users. A sample of 2059 Norwegian women completed a survey reporting: weekly training volume; current HC usage, type, and reasons for use; cycle‐related symptom frequency and severity; and the prevalence of menstrual disorders/disturbances. Respondents were categorized by age (youth: 13–20; young adults: 21–30; older adults: 31–50 years) and training category (minimal: 0; low: < 5; moderate: 5–9; high: ≥ 9 h**·**week^−1^). Half (51.6%) of respondents reported current HC usage, predominantly long‐acting reversible contraception or combined oral contraceptives. Young adults had the highest prevalence of HC use (62.7%) compared to youth (48.4%) and older adults (43.8%), although no differences in usage were seen across training categories. HC users reported fewer and less severe cycle‐related symptoms compared to non‐users. A third (30.8%) of non‐HC users had experienced a menstrual disorder/disturbance, with no significant differences observed across training categories or age groups. In conclusion, HC use is widespread among Norwegian women, with no differences between training categories. This similar HC prevalence suggests that HC research conducted in female cohorts may be generalizable, independent of training category. However, attention should be paid to participants' age due to differences in HC use between age groups.

## 1 Introduction

Since the introduction of the first hormonal contraceptive (HC) in 1960—the combined oral contraceptive pill (COCP) Enovid—different COCP formulations have appeared on the market, as well as progestin‐only contraceptive pills (POCP) and hormonal contraceptives (HCs) using other delivery methods, such as injections, skin patches, vaginal rings, and long‐acting reversible contraception (LARC) in the form of subdermal implants or intrauterine systems (IUS) [1]. In parallel to the increase in the availability of diverse HC types and formulations, a global increase in the percentage of women of reproductive age using contraceptives has been seen [2], with the prevalence of HC use in the general population in 2018 being 40% in Norway [3].

In comparison to the general population, athletes in Norway have a higher prevalence of HC use, with rates ranging from 56% to 68% among (inter)national junior and senior cross‐country skiers and biathletes [4, 5]. Although several studies have included athletes of differing competition categories and/or ages [4, 5, 6, 7, 8, 9], we are currently aware of one study that included a broader spectrum of training categories (i.e., ranging from sedentary women to competitive athletes), as well as women of different age groups [10]. Therefore, the primary objective of this study was to report the point prevalence and type of HC use, along with the reasons for use, among Norwegian women of different training categories and age groups.

Aside from contraception, a commonly reported reason for HC use is to reduce or manage negative symptoms that are perceived to be associated with the menstrual cycle [4, 11, 12, 13]. These symptoms, hereafter referred to as cycle‐related symptoms, encompass symptoms experienced in relation to the menstrual cycle and/or attributed to HC use, while acknowledging that not all HCs are cyclical in nature. Although the types of negative cycle‐related symptoms appear to be similar between HC‐using and non‐using athletes (i.e., abdominal cramps, bloating, mood changes, etc.) [5, 11], the frequency and severity of these symptoms have been suggested to be lower for HC users [4, 8, 11, 13]. To date, we are unaware of studies that separately compare cycle‐related symptom frequency and severity between HC users and non‐users across differing training categories and age groups. Consequently, the secondary aim of the current study was to compare the frequency and severity of cycle‐related symptoms between HC users and non‐users across different training categories and age groups.

In HC users, exogenous hormones alter the functioning of the hypothalamic–pituitary–ovarian axis, whereby the HC hormones influence the extent to which the endogenous ovarian hormones are suppressed and bleeding patterns are influenced [14]. On the other hand, in women not using HC, the menstrual cycle provides feedback about their reproductive health and possibly wider general health [15]. The absence of menses, an irregular menstrual cycle, a short cycle, or a very long cycle may indicate increased levels of physical or psychological stress placed on the body [16]. Although several studies have found an increased prevalence of menstrual disorders (MD) in exercising women compared to sedentary women [17, 18, 19], none of these studies distinguished between different training categories or age groups. It might be expected that women with a high training volume are at higher risk of MD than women with a low training volume because of the increased physical stress placed on the body, as well as that younger women experience more MD due to the immaturity of the hypothalamic–pituitary–ovarian axis [20, 21]. Hence, the third aim was to present the self‐reported bleeding pattern and prevalence of MDs among non‐HC users and investigate the confounding effect of training categories and age groups.

The findings of the current study are expected to provide valuable insights for health professionals, researchers in women's health, coaches, and other members of the support teams for female athletes. By offering a clearer understanding of the prevalence of HC use and the perceived cycle‐related symptoms across different training categories and age groups, this study may assist in guiding informed decision‐making regarding the use and selection of HCs. Furthermore, the data will provide information on the prevalence of MDs among non‐HC users and how this prevalence may vary with training categories and age. Such knowledge could facilitate earlier intervention and the implementation of targeted prevention strategies for at‐risk groups, potentially mitigating the development or progression of MDs.

## 2 Materials and Method

Anonymous data collection was undertaken in two separate collection phases, across an aggregated eight‐month period. From the 1 December 2021 until the 30 April 2022, women in Norway (16–50 years) were invited to anonymously answer a custom designed online questionnaire in Norwegian. This questionnaire collected information on demographics, HC use and type and reasons for usage, frequency and severity of cycle‐related symptoms, and weekly training volume. A second data collection period, focusing on the recruitment of younger school‐aged females (i.e., 13–19 years), took place from the 20 September to the 31 December 2022. Recruitment for the survey occurred via online posts on social media platforms, university intranet, athletic clubs, and word‐of‐mouth. Middle‐ and high schools across Norway were also contacted via email and asked to distribute the survey link to the parents of enrolled students. The same questionnaire was used for both data collection phases and is described in more detail below. Participants were fully informed about the study purpose and provided electronic consent before they were able to access the questionnaire. Inclusion criteria consisted of people who were biologically female and 13–50 years of age. Participants who were younger than 16 years (i.e., 13–15 years) required parental or guardian consent and permission to participate in the project. All respondent data were completely anonymous. The Regional Committee for Medical and Health Research Ethics of Northern Norway (REK Northern Norway) waives the requirement for ethical approval for studies that are not covered by the Health Research Act, and so ethical considerations were performed internally at the university responsible for this research (UiT The Arctic University of Norway). As data were collected anonymously through this project, the Norwegian Agency for Shared Services in Education and Research (Sikt) did not require notification regarding data security, privacy, or data handling.

### 2.1 Questionnaire

The online questionnaire was hosted on Nettskjema, a secure Norwegian survey website developed by the University of Oslo (nettskjema.no). Data were collected using a modified version of previously published questionnaires [4, 5, 22] with alterations based on consultation and feedback from medical experts, coaches, former athletes, sports scientists, and experienced academics. The questionnaire was split into several distinct data collection sections, consisting of: (1) demographic information, (2) current menstrual or HC status, and experience of cycle‐related symptoms over the preceding 12‐months, (3) HC use history, (4) weekly training volume, sport, and athletic performance. Current HC users and non‐HC users completed different portions of section 2, viewing only questions relevant to their group (e.g., menses duration and experience of MDs for non‐HC users, frequency of skipping withdrawal bleed for certain HC users). Symptom frequency was recorded using an ordinal 4‐category response choice (‘never’, ‘a few times per year’, ‘most cycles’, or ‘each cycle’) while symptom severity provided a 5‐category response choice (‘none’, ‘mild’, ‘moderate’, ‘severe’, or ‘extreme’). Respondents reported the number of hours per week engaged in training, sport, and/or planned exercise, with the aggregate of these considered their weekly training volume. Throughout the questionnaire respondents were offered, where possible, the option to provide additional supporting detail and information via use of an open‐ended free text responses. As the questionnaire and results were written in Norwegian, the data was translated to English for analysis by investigators fluent in both languages.

### 2.2 Data Cleaning

**FIGURE 1:**
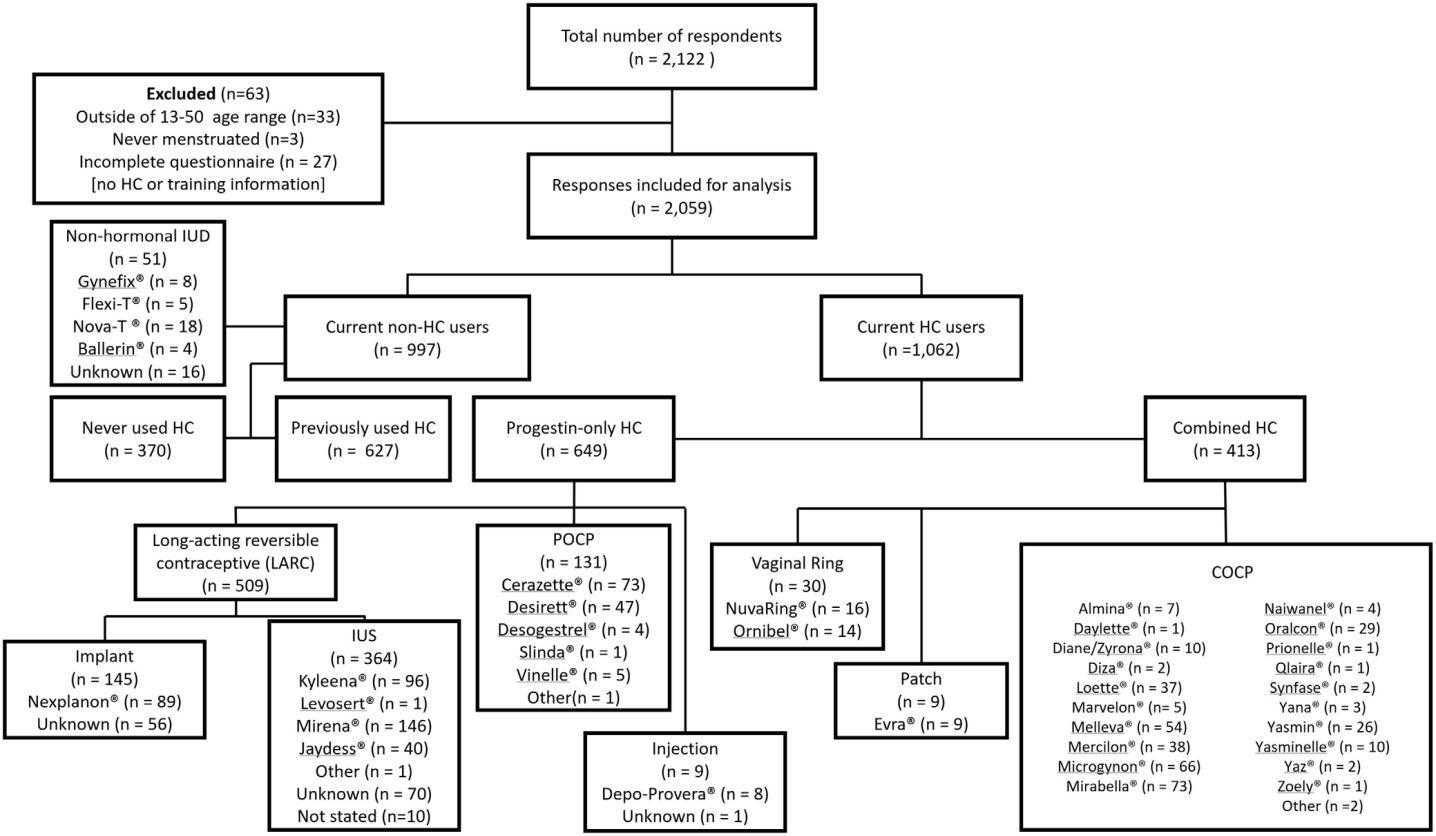
Participant inclusion flowchart and contraceptive brand use. Data presented as frequency. HC, hormonal contraceptive; COCP, combined oral contraceptive pill; IUS, intrauterine system; POCP, progestin‐only contraceptive pill; LARC, long‐acting reversible contraceptive.

As the primary study objective was to describe the point prevalence of HC use in the Norwegian population, inclusion criteria for the participants were: biologically female, aged 13–50 years, had started menstruating, and living in Norway. Data were initially screened to exclude participants who did not meet the inclusion criteria and/or had not completed the required sections of the questionnaire (Figure 1). After this initial screening, participants were grouped into three general age categories: youth (13–20 years); young adults (21–30 years), and older adults (31–50 years). The training category of respondents was classified into four distinct categories based on their self‐reported mean weekly training volume: minimal (i.e., did not engage in regular exercise); low (< 5 h per week of exercise); moderate (5–9 h per week of exercise); and high (≥ 9 h per week of exercise). Previously published criteria were used to classify the ovarian hormone profile, with the length of a ‘regular’ menstrual cycle considered as 21–35 days inclusive [23]. Menstrual disorders (MD) were defined as: no menstruation for three consecutive menstrual cycles (secondary amenorrhea), menstrual cycle length > 35 days (oligomenorrhea), menstrual cycle length < 21 days (polymenorrhoea), heavy or prolonged bleeding (menorrhagia), irregular bleeding/intermenstrual bleeding (metrorrhagia, e.g., bleeding between menses), and severe pain (dysmenorrhea) [21, 24]. The term ‘withdrawal bleeding’ is used henceforth as a blanket term to refer to uterine bleeding experienced by HC users, regardless of HC type.

### 2.3 Data Analysis

Statistical analyses were performed utilizing the R software [25] in the RStudio environment. Binary response data (e.g., HC usage) were modeled via generalized linear regression with a logit link, continuous data (e.g., cycle length) using linear regression, ordinal response data (e.g., symptom frequency) using cumulative (logit) link regression (package: ‘ordinal’) [26] and multinomial data (e.g., HC form/type) using multinomial logistic regression (package: ‘nnet’) [27].

Regression models incorporated fixed factors for group (levels: HC; non‐HC), training category (levels: minimal; 0–4.9; 5–8.9; 9+), age (levels: categorized as 13–20; 21–30; 31–50), along with relevant interaction terms (group by training category by age). As an objective of this study was to investigate symptomology between HC users and non‐users, all analyses for symptom frequency and severity focused exclusively on the comparison between these groups (i.e., HC users and non‐HC), within training categories and age categories, where appropriate. Model fit and diagnostics were checked with the ‘performance’ package [28]. Post hoc testing, effect sizes (i.e., Cohen's *d*; odds ratio [OR]), and estimated marginal means were generated from the ‘emmeans’ package [29]. Multivariate *t*‐distribution adjustment was applied to post hoc testing and statistical significance was set as *α* = 5%. Data are presented as adjusted mean ± standard deviation (SD), frequency (*n*), valid percentages (%), or adjusted odds ratios (OR) with 95% confidence intervals (95% CI).

## 3 Results

### 3.1 HC Prevalence and Reasons of Use

A total of 2059 respondents were included in the final analysis (Figure 1), with approximately half currently using HCs (51.6%; Table 1). Anthropometric data can be viewed in Table S1.

**TABLE 1:** Hormonal contraceptive use prevalence in Norwegian women, stratified by age and self‐reported weekly training volume.

	Overall	Self‐reported weekly training volume (sport, exercise, or training)			
	*n* = 2059	Minimal	Low (< 5 h)	Moderate (5 to 9 h)	High (≥ 9 h)
Demographic information					
Age group (years)					
All combined (13–50)	2059	792	320	522	425
13–20	577	239	59	98	181
21–30	708	242	103	211	152
31–50	774	311	158	213	92
Hormonal contraceptive usage					
Non‐users					
All combined (13–50)	997 (48.4%)	404 (51.0%)	151 (47.2%)	237 (45.4%)	205 (48.2%)
13–20	298	122	29	52	95
21–30	264	10	34	72	56
31–50	435	180	88	113	54
Current HC users					
All combined (13–50)	1062 (51.6%)	388 (49.0%)	169 (52.8%)	285 (54.6%)	220 (51.8%)
13–20	279 (48.4%)	117	30	46	83
21–30	444 (62.7%)^a^, ^b^	140	69	139	96
31–50	339 (43.8%)	131	70	100	38

*Note:* Data presented as frequency (valid % of group within age category and training group).

Abbreviation: HC, hormonal contraceptive.

^a^ Indicates significantly different to 21–30.

^b^ Indicates significantly different from 31 to 50.

No significant main effect of training category (*p* = 0.393) was found for the prevalence of HC usage; however, there was an effect of age group (*p* < 0.001). Post hoc analysis indicated that young adults (21–30 years old) were more likely to use HC compared to youth (13–20 years old; *p* < 0.001; OR = 1.77; 95% CI = 1.35, 2.32) and older adults (31–50 years old; *p* < 0.001; OR = 2.16; 95% CI = 1.68, 2.78). There was no significant interaction of training category by age group (*p* = 0.837) for HC usage.

The majority of HC users reported currently using either COCP (35.2% of users; *n* = 374) or IUSs (34.2%; *n* = 363), followed by the subdermal implant (i.e., ‘implant’; 13.7%; *n* = 145) and POCP (12.4%; *n* = 132). Relatively few respondents reported using the vaginal ring, contraceptive injection, or contraceptive patch (see Table 2). Age was associated with the type of HC being used (*p* < 0.001); however, no difference was observed between training categories (*p* = 0.627) or age by training interaction (*p* = 0.404). The older adults reported lower use of COCP (23.0%) and implants (6.2%) when compared to other age categories (youth: 40.1% [COCP], 21.9 [implant]; young adult: 41.4% [COCP], 14.2% [implant]). The proportion of IUS users increased with age, rising from 14.7% among youth to 30.9% among young adults, and reaching over half (54.6%) of the HC‐using older adults.

**TABLE 2:** Type of hormonal contraceptive type used by Norwegian women, stratified by self‐reported weekly training volume and age group.

Type of hormonal contraceptive	HC Users	Self‐reported weekly training volume (sport, exercise, or training)			
	*n* = 1062	Minimal	Low (< 5 h)	Moderate (5 to 9 h)	High (≥ 9 h)
All HC types combined	1062	388	169	285	220
Combined oral contraceptive pill (COCP)	374 (35.2%)	139 (35.8%)	49 (29.0%)	104 (36.5%)	82 (37.3%)
Intrauterine system (IUS)	363 (34.2%)	125 (32.2%)	71 (42.0%)	107 (37.3%)	60 (27.3%)
Contraceptive implant	145 (13.7%)	58 (14.9%)	20 (11.8%)	26 (9.1%)	41 (18.6%)
Progestin‐only contraceptive pill (POCP)	132 (12.4%)	47 (12.1%)	20 (11.8%)	37 (13.0%)	28 (12.7%)
Vaginal ring	30 (2.8%)	11 (2.8%)	6 (3.6%)	7 (2.5%)	6 (2.7%)
Contraceptive injection	9 (0.8%)	5 (0.6%)	1 (0.6%)	3 (1.0%)	0
Contraceptive patch	9 (0.8%)	3 (0.4%)	2 (1.2%)	1 (0.4%)	3 (1.4%)

Type of hormonal contraceptive	HC Users	Age group (years)		
	*n* = 1062	13–20	21–30	31–50
All HC types combined	1062	279	444	339
Combined oral contraceptive pill (COCP)	374 (35.2%)	112 (40.1%)^a^	184 (41.4%)^a^	78 (23.0%)
Intrauterine System (IUS)	363 (34.2%)	41 (14.7%)^a^, ^b^	137 (30.9%)^a^	185 (54.6%)
Contraceptive implant	145 (13.7%)	61 (21.9%)^a^	63 (14.2%)^a^	21 (6.2%)
Progestin‐only contraceptive pill (POCP)	132 (12.4%)	56 (20.1%)^a^, ^b^	41 (9.2%)	35 (10.3%)
Vaginal ring	30 (2.8%)	3 (1.1%)	12 (2.7%)	15 (4.4%)
Contraceptive injection	9 (0.8%)	2 (0.7%)	3 (0.7%)	4 (1.2%)
Contraceptive patch	9 (0.8%)	4 (1.4%)	4 (0.9%)	1 (0.3%)

*Note:* Data presented as frequency (valid % of column group).

Abbreviations: HC, hormonal contraceptive; COCP, combined oral contraceptive pill; IUS, intrauterine system; POCP, progestin‐only contraceptive pill.

^a^ Indicates significantly different from 31 to 50.

^b^ Indicates significantly different to 21–30.

The primary reason for using HC was contraception (69.8% of users), with 30.2% reporting a different reason, such as symptom management. No difference was seen between training categories. There was a significant effect of age on the reason for HC usage, with a higher likelihood of older adults using HC primarily for contraception, compared to youth (*p* = 0.030; OR = 3.95). On average, respondents had continuously used their current HC for 4.0 ± 3.9 years (range: < 1 month to more than 23 years), with 64% (*n* = 680) having previously used a different HC. The reasons for changing or discontinuing the previous HC brand and/or type varied, such as side effects (50.3% of HC users that had previously used a different HC), ease of use (9.9%), trying to get pregnant (6.1%), no longer needing contraception (5.9%), or on recommendation from health professionals (3.1%).

Approximately two thirds (62.9%) of non‐HC users had previously used some form of HC, primarily COCP or POCP (72.9% of group), followed by subdermal implant (13.0%) and IUSs (6.9%). Youth were significantly less likely to have previously used HCs compared to both young adults (*p* < 0.001; OR = 0.04; 95% CI: 0.02, 0.07) and older adults (*p* < 0.001; OR = 0.03; 95% CI: 0.02, 0.04), while training category showed no significant effect (*p* = 0.717). The predominant reason cited for discontinuing HC use was side effects, reported by 42.9% of this group (i.e., 27% of all non‐HC users), with mood changes followed by weight gain reported as the most common side effects.

### 3.2 Frequency and Severity of Cycle‐Related Symptoms in HC and Non‐HC Users

The majority of respondents (*n* = 1670; 81.1%) reported at least one negative symptom during most, or every, HC cycle or menstrual cycle. The most commonly reported symptoms included bloating (56.4%), mood changes (54.9%), cramps (51.8%), and fatigue (47.7%). Only 1.2% of non‐HC users reported that they never experienced cycle‐related symptoms, compared to 17.2% of HC users (*p* < 0.001; OR = 16.0; 95% CI: 8.3–30.8).

**FIGURE 2:**
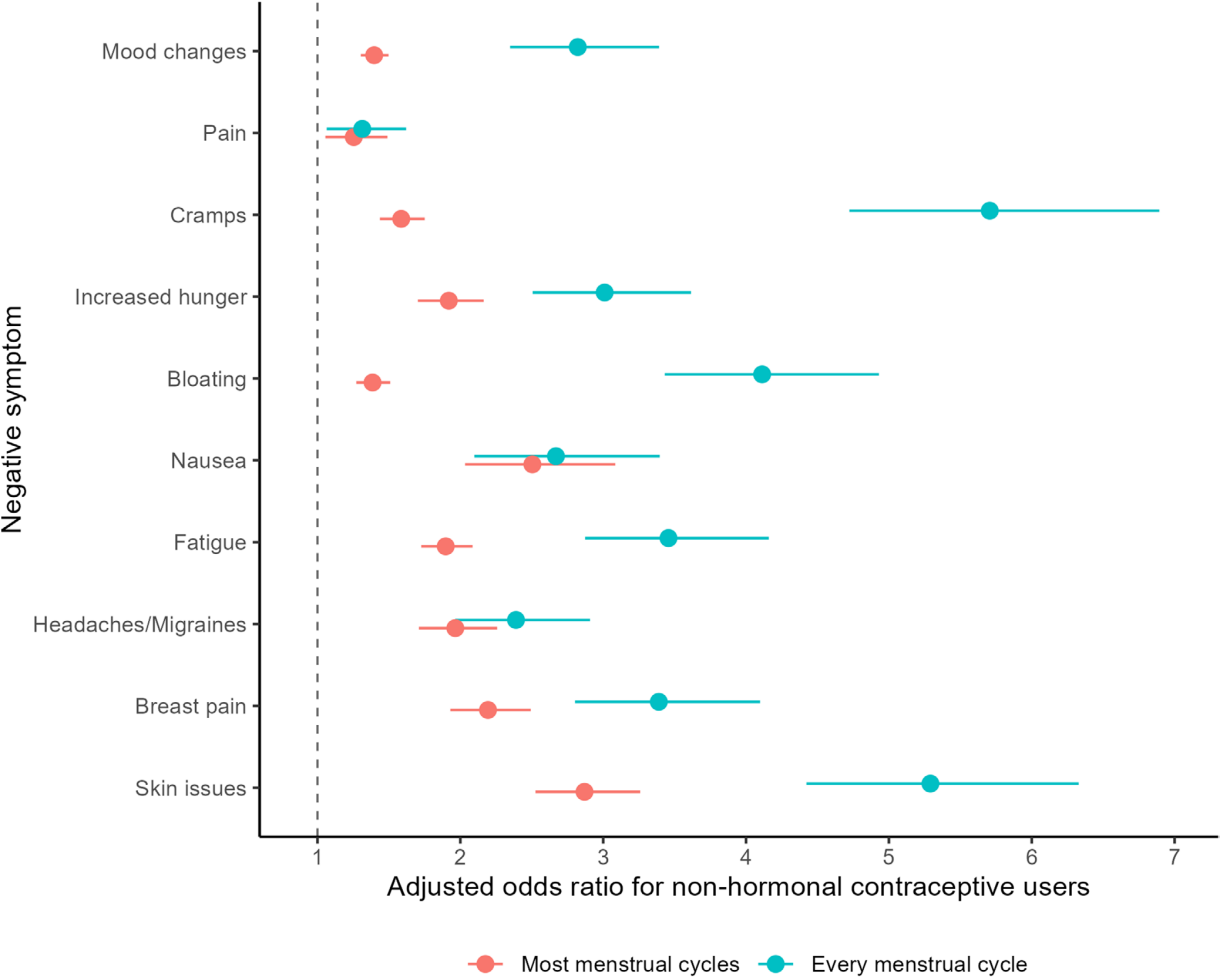
Adjusted odds ratio (OR) and 95% confidence intervals for the occurrence of self‐reported negative menstrual cycle‐related symptoms by non‐hormonal contraceptive (non‐HC) users. The dotted vertical line at OR = 1 represents the reference HC group.

Compared to HC users, non‐HC users had significantly higher odds of reporting the occurrence of skin problems (*p* < 0.001; OR = 2.9–5.3), headaches (*p* < 0.001; OR = 2.0–2.4), sore breasts (*p* < 0.001 to 0.007; OR = 2.2–3.4), fatigue (*p* < 0.001; OR = 1.9–3.5), bloating (*p* < 0.001; OR = 1.4–4.1), hunger (*p* < 0.001; OR = 1.9–3.0), cramps (*p* < 0.001; OR = 1.6–5.7), pain (*p* = 0.010–0.011; OR = 1.3–1.3) and mood changes (*p* < 0.001–0.006; OR = 1.1–3.2), most or every cycle (Figure 2) for all training categories and age groups.

A significantly lower proportion of HC users (16.4%), regardless of training category or age group, needed medication to treat and/or manage their cycle‐related symptoms, when compared to non‐HC users (34.8%; *p* = 0.009, OR = 0.48; 95% CI: 0.40, 0.59). However, no difference was seen in medication usage between different types of HC (*p* = 0.192). More than three‐quarters (76.5%) of COCP users reported that they have deliberately ‘skipped’ a withdrawal bleed (i.e., continuing to take active pills), mainly to avoid abdominal cramping or other cycle‐related symptoms, general inconvenience (e.g., traveling), and/or to avoid interfering with athletic training/sport.

Compared to HC users, non‐HC users had significantly higher odds of reporting moderate‐to‐extreme symptom severity for skin problems (all *p* < 0.001; OR = 1.26–1.70), headaches (*p = 0*.002–0.005; OR = 1.11–1.45) and cramps (*p* < 0.001–0.006; OR = 1.08–2.02). No differences were found for breast pain (*p* = 0.860), nausea (*p* = 0.100), bloating (*p* = 0.105), hunger (*p* = 0.053) and pain (*p* = 0.656).

A higher likelihood of moderate‐to‐extreme fatigue severity was reported by non‐HC users compared to HC users for young adults (*p* = 0.001–0.005; OR = 1.12–1.78) and older adults (*p* < 0.001–0.006; OR = 1.25–2.17), but no difference was seen for youth (*p* = 0.116). Similarly, non‐HC users were more likely to report higher moderate‐to‐extreme symptom severity for hunger among young adults (*p* = 0.005–0.009; OR = 1.19–1.77) and severe‐to‐extreme symptom severity among older adults (*p* = 0.033–0.031; OR = 1.55–1.65) but with no significant differences in youth (*p* = 0.599).

### 3.3 Self‐Reported Bleeding Patterns and Prevalence of Menstrual Disturbances in Non‐HC Users

Approximately half of all respondents (48.4%; *n* = 997) reported not using HCs at the time of completing the questionnaire, with 5.1% (*n* = 51) of this group currently using non‐hormonal copper intrauterine devices. The majority (78.8%) of non‐HC respondents self‐reported an average cycle length between 21 and 35 days over the past 12 months, with a mean cycle length of 29.3 ± 6.7 days. No differences in cycle length were seen between training categories (*p* = 0.953). Older adults had a significantly shorter average cycle length (28.4 days; 95% CI: 27.7, 29.1) compared to youth (*p* = 0.023; *d* = 0.24; 30.0 days; 95% CI: 29.0, 30.9) and young adults (*p* = 0.005; *d* = 0.28; 30.2 days; 95% CI: 29.3, 31.2). Typical menses length was 5.3 ± 1.7 days, with a cycle‐to‐cycle variation of approximately 2 days. No effect of training category or age was shown for menses length (*p* = 0.369–0.372). Various MDs were self‐reported by 30.8% of non‐HC users, including secondary amenorrhea (*n* = 34), oligomenorrhea (*n* = 112), polymenorrhea (*n* = 26), heavy or prolonged bleeding (i.e., menorrhagia, *n* = 101), irregular bleeding (i.e., metrorrhagia, *n* = 69), and/or severe pain (i.e., dysmenorrhea, *n* = 55). Several participants reported experiencing multiple MDs (e.g., oligomenorrhea and severe pain). No difference in the likelihood of any MDs, or form of MD, was found between training categories, age, or the interaction of these factors.

## 4 Discussion

The present study aimed to (1) report the prevalence, type, and reasons for HC use among Norwegian women of different training categories and age groups, (2) compare the frequency and severity of self‐reported cycle‐related symptoms, and (3) describe the self‐reported bleeding patterns and prevalence of MDs among non‐HC users and investigate the confounding effects of training categories and age groups. Just over half (51.6%) of the total sample reported currently using HC, primarily LARCs (i.e., IUSs or implants) or COCPs, with no significant differences in usage rates between training categories. Young adults were more likely than youth or older adults to use HCs, and usage of IUSs increased with age. Although most respondents (69.8%) noted that contraception was the primary reason for HC usage, management of adverse cycle‐related symptoms was also a prevalent reason. Compared to HC users, non‐HC users had a higher likelihood of more frequent and severe negative cycle‐related symptoms and used medication to treat or manage these symptoms. At the time of answering the questionnaire, 30.8% of non‐HC users reported experiencing some form of MD; however, neither training category nor age group significantly influenced the likelihood of MD prevalence. Overall, the present study found similar HC prevalence across different training categories (within the same country), suggesting that HC‐related outcomes from one training population are possibly applicable to other training populations. However, the variation in HC prevalence between age groups highlights the importance of considering participant age when interpreting female‐specific data, as differing HC usage rates may influence results.

### 4.1 HC Prevalence and Reasons of Use

The use of HCs (51.6%) was widespread across the current cohort and considerably higher than reported earlier in a nationwide Norwegian register‐based study, where the prevalence of HC usage between 2006 and 2018 was ~34%–40% in the general population [3]. The longitudinal data from Furu et al. [3] highlighted a trend of increasing HC use in recent years and, if extrapolated, would approximately align with the current study prevalence. Our prevalences are similar to the prevalence (~48%) reported for a group of Norwegian women aged 40–49 years (2015–2016) [30] as well as the prevalence in a group of regional to international level Norwegian handball players (47%) [31], but lower than the rates for (inter)national level Norwegian endurance athletes (56%–68%, 2018–2020) [4, 5]. The ~50% HC usage observed in the present study is also relatively high when contrasted against other Western countries, such as the UK (37.5%) [32], Spain (~30%) [33], and the USA (27.5%) [34]. The higher prevalence of HC use in the present Norwegian cohort highlights the importance of considering HC use when studying health and performance outcomes in active women, as it may influence physiological responses, symptom profiles, and broader health trends [4, 5, 35]. The variations in HC usage between countries suggest that factors beyond individual choice—such as healthcare policies, accessibility, and cultural attitudes—may play a role in shaping HC usage patterns.

HC usage appeared to be independent from weekly training volume, with all training categories reporting ~50% HC usage (ranging from 49.0% to 54.6%), and no differences between training categories for the type of HC used. This ~50% HC prevalence is similar to the usage rates in several earlier studies on athletes from various countries and sports (45%–57%) [5, 12, 13, 22, 31], but slightly higher than observed in mixed‐sport Australian and UK athlete cohorts (41%–42%); [6, 8] and somewhat lower than recent reports in Swedish and Norwegian athletes (63%–68%) [4, 7]. Previous research has also noted a possible decrease in HC usage with higher competition categories [7, 8]. Such discrepancies are potentially due to the focus on high competition level (e.g., national to world‐class athletes) in these samples, compared to the present study where the ‘high’ training category (i.e., ≥ 9 h weekly training, sport, and exercise) likely also included many recreational‐level athletes. The observed consistency in HC usage across different training categories suggests that HC choice may be relatively unaffected by volume of training, at least within the sample studied. This finding can help inform coaches and practitioners, highlighting that factors other than training volume, such as personal reproductive health goals, might play a larger role in the HC usage decisions. It also implies that interventions targeting HC use could be beneficial across a broad range of athletes, regardless of their training volume or competition level.

Young adults were more likely to use HCs (62.7%), compared to youth or older women (48.4% and 43.8%, respectively), which aligns with similar data from earlier Norwegian research [3, 36]. Reasons for these age‐based differences potentially stem from an increased need for sexual contraception, as only half (56.6%) of youth HC users reported contraception as the primary reason for HC usage, compared to 74.1%–74.9% of both adult HC user groups, which is similar to the proportion recently reported for young adult athletes (82%); [7]. For older adults, the lower overall HC usage may also reflect an increased focus on achieving pregnancy, as the average age of first‐time mothers is reported to be 30.3 years in Norway [37]. These age‐related differences in HC usage highlight the importance of considering a tailored approach when working with athletes of different age groups. For example, older athletes may be more likely to shift away from HC use as their reproductive goals change, altering the occurrence and severity of cycle‐related symptoms. Understanding these age‐associated patterns in HC use may help coaches and sports practitioners better support female athletes by aligning training and performance strategies with their contraceptive choices and symptomology.

Previous nationwide Norwegian data has indicated a rise in the usage of LARCs (i.e., IUSs and subdermal implants) over the preceding decade, concurrent with a decrease in COCP [3]. However, the present study appears to be one of the first to report LARCs as the most prevalent type of HC (47.8%) by Norwegian women of different ages (from youth to older adults; 13–50 years) and training categories, followed by COCP (35.2%). Analysis of free‐text responses from respondents who had switched from COCPs to LARCs highlighted that ‘convenience’ was a common reason for the change, specifically not needing to remember to take the pill daily. Several other factors have also likely contributed to the observed change in the populator of LARC usage. For example, all types of LARCs have been subsidized by a Norwegian governmental reimbursement scheme since 2015, reducing or eliminating the financial burden associated with HC use [38]. Simultaneously, the Norwegian Medicines Agency recommended LARCs as the optimal choice for new HC users, while from 2016, public health nurses and midwives were permitted to prescribe LARCs and other HC types to Norwegian women, including a focus on younger ages, increasing HC accessibility [3, 38]. The rise in LARC usage, driven by factors like convenience, government subsidies, and improved accessibility, highlights a shift in contraceptive preferences among Norwegian women, offering valuable insight for healthcare providers and policymakers in shaping future contraceptive recommendations.

### 4.2 Frequency and Severity of Cycle‐Related Symptoms in HC and Non‐HC Users

The majority of respondents (81%), regardless of HC use, reported regularly experiencing at least one negative cycle‐related symptom, such as bloating or mood changes, during most or every menstrual cycle or as the result of HC usage, which is consistent with previous findings [6, 39, 40]. Non‐HC users were markedly more likely than HC users to report a frequent occurrence for all possible negative symptom options that were available in the survey, as illustrated in Figure 2, regardless of training category or age group. HC use is known to reduce negative symptom prevalence [7] and frequency [8] for both the general population and athletes [6, 7, 8, 41]. Mechanistically, the exogenous hormones from HC have been suggested to stabilize endogenous hormonal fluctuations and reduce symptom occurrence and/or severity [42]. Indeed ~30% of HC users in the current study noted that their primary reason for usage was non‐contraceptive, with many COCP users deliberately skipping inactive pills or the pill‐free week to avoid negative cycle‐related symptoms, mirroring the results of earlier studies which reported ovarian hormone profile manipulation by COCP users to attenuate symptoms [7, 22, 43]. Furthermore, the cessation of bleeding when using LARCs [4] might partly explain the lower symptom frequency in HC users. Likewise, HC users were less likely to use medication to manage negative symptoms compared to non‐HC users (OR = 0.48), potentially supporting that HC users may have a lower frequency and/or severity of negative symptoms. Nonetheless, it is important to acknowledge that a quarter (27%) of non‐HC users in the present sample had previously discontinued HC due to adverse side effects, underscoring the potential for negative individual responses to exogenous hormone administration and the necessity of personalized approaches to menstrual symptom management.

Non‐HC users reported significantly higher odds of experiencing more severe negative cycle‐related symptoms, including skin problems, headaches, and cramps, across all training categories and age groups compared to HC users. However, differences in the severity of fatigue and hunger between HC and non‐HC users were selectively observed only among young and older adults, with no significant differences identified in youth. While previous research has linked increased frequency of food cravings and fatigue associated with the menstrual cycle to increasing age [44], the severity of these symptoms has not been previously assessed. Although the present data suggest that HC usage may moderate the severity of fatigue and hunger in certain age groups, further evidence is needed to clarify the potential underpinning mechanisms and to better understand how HC type may influence food cravings and fatigue across different age groups. The lack of significant differences in severity for nausea, bloating, hunger, and pain between HC users and non‐HC users suggests these symptoms may either be less responsive to hormonal modulation or influenced by other unmeasured factors such as lifestyle or environmental variables.

### 4.3 Self‐Reported Bleeding Patterns and Prevalence of Menstrual Disturbances in Non‐HC Users

Nearly one‐third (30.8%) of non‐HC users self‐reported experiencing a MD, without influence from training category and/or age group. The prevalence of oligomenorrhea (11.2%) and amenorrhea (3.4%) falls somewhere between the rates reported for sedentary (0% for both) and exercising women (37.2 and 7.0%, respectively) [19], or competitive athletes (23.5% and 7.1%, respectively) [21]. However, the absence of an effect of training category on MD prevalence is surprising, given previous data indicating a higher rate of MDs in exercising women compared to their sedentary counterparts [19], as well as the tendency for a higher prevalence of secondary amenorrhea and oligomenorrhea in higher‐caliber athletes [21]. This difference might be due to the self‐report nature of the data in the current study compared to the observational prospective study conducted by De Souza et al. [19]. Additionally, the different athlete caliber groups (tier 2 vs. tier 3 vs. tier 4) in the systematic review of Taim et al. [21] were likely combined into the singular ‘high’ training group of the current study. Similarly, the absence of a confounding effect of age group is unexpected, since a higher prevalence of severe MD in adolescent athletes compared to adult athletes has been previously reported [21]. However, Taim et al. [21] pooled data from multiple studies in their review, thereby amplifying the sample size and statistical power to detect differences. Further, the age groupings in the present study are relatively broad, with the youth group (13–20 years) encompassing both young teenagers who may have only recently reached menarche, as well as older teenagers with more stable menstrual cycles. Distinct effects of training category and/or age group might have emerged with more defined groups and with a larger sample size per group.

### 4.4 Strength and Limitations

While previous research also included young Norwegian women as participants (16–49 or 15–49) [3, 36], this cross‐sectional study is the first to report the prevalence and types of HC use, as well as the self‐reported cycle symptom frequency and severity of cycle‐related symptoms within this population. Further, the present study's inclusion of different training categories and age groups permitted symptomology comparison and investigation of potential differences due to these factors. However, several limitations should be noted. As the study was cross‐sectional, it only provides data at a single timepoint and limits the possibility of investigating causality and changes over time. Study participation may have been affected by self‐selection bias, as women with a pre‐existing interest in HC usage and/or cycle‐related symptomology may have been more receptive to participate, potentially skewing the data. Recruitment occurred primarily via social media (phase 1), which may have limited the demographic and thus not provided a representative sample of the population, potentially limiting the generalizability of the results. In addition, perimenopausal or menopausal women were not specifically excluded from this study, which may have confounded some results, particularly around symptomology in the older age group. It should also be acknowledged that various types of HCs (e.g., intrauterine systems (IUS), combined oral contraceptive pills (COCP), etc.) have been associated with differing effects on cycle‐related symptoms. Consequently, grouping all HC types together in the analysis may have obscured specific effects attributable to individual types of contraceptives.

## 5 Perspectives

The present study found that approximately half of the surveyed Norwegian women used some form of HC, primarily for avoiding pregnancy, with the highest usage rates reported among young adults. This relatively high prevalence of HC usage, regardless of training categories, underscores the importance of accounting for HC status when investigating health or training adaptations in physically active women [7]. The predominant HC delivery methods were either COCP or LARCs, similar to previous Norwegian data [3, 4], with LARC usage found to be more prevalent as user age increased. Weekly training volume was not associated with differences in HC prevalence. Compared to HC users, non‐HC users were more likely to report frequent and severe cycle‐related symptoms and to use medication to treat these symptoms, supporting the role of HCs in potentially mitigating cycle‐related symptoms [4, 5, 8]. Given the challenges of training while symptomatic [6], these findings highlight possible opportunities for tailored symptom management strategies (e.g., proactive education) between support teams and athletes with severe symptomology.

## Author Contributions

Study conceptualization and piloting: J.O.O., T.P.E., E.P.A., G.S.S., Ø.S., B.M., K.J.E.S., and B.W. Data collection and cleaning: J.O.O. and J.H.S. Analysis: J.O.O. First draft: J.O.O. and D.A.N. Reviewing final manuscript: all authors.

## Ethics Statement

All data were collected anonymously, and participants consented to participate in the study prior to completing the survey form. The Norwegian Agency for Shared Services in Education and Research (Sikt) did not require notification regarding data security, privacy, or data handling, due to the anonymous nature of the data collection.

## Consent

The authors have nothing to report.

## Conflicts of Interest

The authors declare no conflicts of interest.

## Supporting information


**Table S1.** Anthropometric data stratified by age and self‐reported weekly training volume.

## Data Availability

The datasets used and analyzed during the current study are available from the corresponding author on reasonable request.
