# Supplementary material for: Prevalence of Hormonal Contraceptive Use and Self‐Reported Symptomatic Experiences Attributed to the Menstrual Cycle or Hormonal Contraceptive Use in Norwegian Women: The Effect of Training Categories and Age Groups ‐ The FENDURA Project
Source: Scand J Med Sci Sports. 2025 Jul 12;35(7):e70096. doi: 10.1111/sms.70096 (PMC12254879; doi:10.1111/sms.70096)
Supplement: Supplementary file 1 — Table S1. Anthropometric data stratified by age and self‐reported weekly training volume. [file SMS-35-e70096-s001.docx]

**Supplementary Table 1. Anthropometric data stratified by age and self-reported weekly training volume.**

| Variables |  | Overall | Self-reported weekly training volume (sport, exercise, or training) | | | |
| --- | --- | --- | --- | --- | --- | --- |
|  | **Age (years)** | **n = 2,059** | **Minimal** | **Low (<5 h)** | **Moderate (5 to 9 h)** | **High (≥9 h)** |
| Height (cm) |  | 167 ± 7 | 167 ± 8 | 168 ± 7 | 168 ± 6 | 168 ± 6 |
|  | 13–20 | 168 ± 7 | 167 ± 7 | 167 ± 7 | 168 ± 6 | 168 ± 6 |
|  | 21–30 | 168 ± 8 | 167 ± 10 | 168 ± 7 | 168 ± 6 | 169 ± 6 |
|  | 31–50 | 168 ± 6 | 168 ± 6 | 168 ± 6 | 168 ± 6 | 169 ± 7 |
| Body mass (kg) |  | 68.4 ± 14.0 | 70.7 ± 16.8 | 67.1 ± 12.1 | 67.0 ± 11.7 | 67 ± 11.4 |
|  | 13–20 | 62.6 ± 11.0 | 62.7 ± 13^#,$^ | 59.3 ± 8.9^#,$^ | 63.6 ± 11.2^$^ | 62.9 ± 8.4^#,$^ |
|  | 21–30 | 68.8 ± 14.0 | 71.3 ± 17.9^$,†,§^ | 66.9 ± 8.6 | 66.3 ± 11.4 | 69.7 ± 12.0 |
|  | 31–50 | 72.2 ± 14.5 | 76.2 ± 16.1^†,§,‡^ | 70.3 ± 13.7 | 69.2 ± 11.7 | 69.3 ± 13.1 |

Data presented as frequency (valid % of group). Within the same training category: ^#^ indicates significantly different to ‘21–30’; ^$^ indicates significantly different to ‘31–50’. Within the same age category: ^†^ indicates significantly different to *Low*; ^§^ indicates significantly different to *Moderate*, and ^‡^ indicates significantly different to *High*.
